# Ectopic pregnancy: search for biomarker in salivary proteome

**DOI:** 10.1038/s41598-023-43791-7

**Published:** 2023-10-06

**Authors:** Archunan Priya Aarthy, Sangeetha Sen, Mahalingam Srinivasan, Subramanian Muthukumar, Pakirisamy Madhanraj, Mohammad Abdulkader Akbarsha, Govindaraju Archunan

**Affiliations:** 1 Department of Obstetrics and Gynecology, Rabindra Nath Tagore Medical College, Udaipur, Rajasthan India; 2 Department of Obstetrics and Gynecology, Saveetha Medical College and Hospital, Deemed University, Chennai, India; 3 Department of Animal Science, Bharathidasan University, Tiruchirappalli, Tamil Nadu India; 4 Deparment of Biotechnology, School of Chemical & Biotechnology (SCBT), SASTRA Deemed University, Thanjavur, Tamil Nadu India; 5 Department of Microbiology, Marudupandiyar College, Thanjavur, Tamil Nadu India; 6 Mahatma Gandhi-Doerenkamp Centre for Alternatives, Bharathidasan University, Tiruchchirappalli, India; 7 Department of Biotechnology & Microbiology, National College (Autonomous), Tiruchchirappalli, India; 8 Marudupandiyar College, Thanjavur, Tamil Nadu India

**Keywords:** Predictive markers, Predictive markers, Molecular medicine

## Abstract

Ectopic pregnancy (EP) is associated with high maternal morbidity and mortality. Ultrasonography is the only dependable diagnostic tool for confirming an ectopic pregnancy. In view of inadequate early detection methods, women suffer from a high-life risk due to the severity of EP. Early detection of EP using pathological/molecular markers will possibly improve clinical diagnosis and patient management. Salivary proteins contain potential biomarkers for diagnosing and detecting various physiological and/or pathological conditions. Therefore, the present investigation was designed to explore the salivary proteome with special reference to EP. Gel-based protein separation was performed on saliva, followed by identification of proteins using Liquid Chromatography-Tandem Mass Spectrometry (LC–MS/MS). Totally, 326 proteins were identified in the salivary samples, among which 101 were found to be specific for ruptured ectopic pregnancy (EPR). Reactome analysis revealed innate immune system, neutrophil degranulation, cell surface interactions at the vascular wall, and FCERI-mediated NF-kB activation as the major pathways to which the salivary proteins identified during EPR are associated. Glutathione-S-transferase omega-1 (GSTO1) is specific for EPR and has been reported as a candidate biomarker in the serum of EPR patients. Therefore, saliva would be a potential source of diagnostic non-invasive protein biomarker(s) for EP. Intensive investigation on the salivary proteins specific to EP can potentially lead to setting up of a panel of candidate biomarkers and developing a non-invasive protein-based diagnostic kit.

## Introduction

Ectopic pregnancy or extrauterine pregnancy (EP) is a condition in which the implantation and development of the fertilized ovum occurs outside to the uterus. In most of the EP cases implantation occurs in the fallopian tube, which is referred to as tubal pregnancy. In some cases non-tubal ectopic pregnancy occurs in which the implantation occurs in the ampulla, ovary, cervix, peritoneal cavity, etc.^1^. Moreover, EP is considered as a serious problem since it is known to lead to maternal mortality and morbidity^2–4^. The highest incidence of EP has been recorded in the African continent^5^. In America the rate of EP has been increasing dramatically over the past decade^6^. In India the incidence of maternal death due to ectopic pregnancy, in a 6 year retractive study, was 3.5–7%^7^. The incidence is more common in women aged 26–30 years^8^. According to a recent report there is increased incidence of EP due to COVID-19 infection^9^, the reason for which is that it could not be diagnosed based on the symptoms of abdominal pain (high blood flow in abdomen) and/or vaginal bleeding. The clinical diagnosis of EP starts with measurement of serum βhCG and progesterone, but ultrasound imaging is possibly the reliable diagnostic method. Depending on the severity of the condition, methotrexate treatment or surgical procedure is the possible clinical management practice for EP patients.

Human saliva plays a vital role in food ingestion, digestion, and oral health. Saliva is one of the few biological fluids in humans that offers a potential non-invasive source of biomolecules to represent the physiological statuses and disease conditions. Saliva contains many electrolytes, proteins (i.e., mucins, enzymes, and immunoglobulins), lipids, hormones, and other molecules^10^. Human saliva, put together, contains more than 3652 proteins and 12,562 peptides, accounting for 51% of proteins, and 79% of plasma peptides, respectively^11,12^. The salivary proteome analysis reveals that amylase and mucin constitute a major portion of the proteins^13^, but the concentration varies from person to person^14^.

Over the past decade, exploration of body fluid proteins has been considered as one of the plausible approaches to find candidate biomarkers for various diseases in the humans^15–18^. Thus, salivary proteins have been recommended as biomarkers to predict disease conditions such as Sjogren’s syndrome^19^, lung cancer^20^, oral cancer^21^, several systemic diseases^22^, HIV infection^23,24^, dental pellicle development^25^, hyperglycemia^26^, etc. Recently, it has been reported that salivary hormones and proteins have the potential to predict ovulation in women^27,28^. Saliva offers advantages such as easy non-invasive sampling, potential for large-scale studies, and the development of point-of-care platforms. Therefore, efforts are being put to compare saliva and serum/plasma composition for disease monitoring^29^. Additionally, salivary proteome analysis has provided valuable insights into the pathology of Type 1 diabetes in a pediatric population^30^, served as a fluid signature for inflammatory and immune-mediated skin diseases^31^, revealed transition signatures from healthy to periodontal disease^32^, and offered specific signatures in the early and middle stages of human pregnancy with term birth outcome^33^.

Currently, only the serial quantitative assay of hCG and transvaginal ultrasound are the major diagnostic methods for EP^34^. Although these tools have enhanced the clinician’s ability to detect the disease, repeated evaluation is often required. Nevertheless, early diagnosis still remains a challenge, causing delay in disease management^35^. By invasive procedures, several biochemical markers have been studied and used for diagnosis by clinicians which include activin A, activin B, inhibin A, follistatin, A disintegrin and metalloprotease-12 (ADAM), pregnancy-associated plasma protein A (PAPP-A), pregnancy-specific B1-glycoprotein (SP1), interleukins 6 and 8 (IL-6 and IL 8), placental-like growth factor (PGF), vascular endothelial growth factor (VEGF), glycodelin (Glyc), etc.^36–42^. The ratio of EP to live births has been shown to increase with maternal age, and this ratio increased significantly from 11.0 to 13.7 ectopic pregnancies per 1000 live births between 2006 and 2013^6^. Barnhart^43^ found that about half the percentage of people diagnosed with EP do not possess identifiable risk factors, including sexually transmitted infections, tubal damage, and pelvic inflammatory disease. Defects in ciliary movement and/or muscular contractions of fallopian tube, which facilitate transportation of embryo caused by infection or smoking, are the possible pathogenic mechanism for tubal ectopic pregnancy^44^. Reduction in adrenomedullin, a receptor protein, is a major factor that impairs embryo transport in fallopian tube and, thus, leads to tubal EP^45,46^. Many molecular approaches, including proteomics, have been explored for biomarker discovery for ectopic pregnancy. In a recent study, GSTO1 has been identified as a favorable protein biomarker from serum^47^. From the perspective of non-invasive approach, there has been no critical lead for salivary protein biomarker(s) for EP. Thus, we hypothesized that one or more salivary proteins would be potential non-invasive biomarker(s) to diagnose ectopic pregnancy. Therefore, this study attempted to explore the profile of salivary proteins during ectopic pregnancy by adopting gel-based protein mass spectrometry.

## Materials and methods

### Sample collection and processing

After obtaining proper informed consent from the volunteers, the saliva samples were collected as per the protocol approved by the Institutional Human Ethical Committee of Ravindra Nath Tagore (RNT) Medical College, Udaipur, India. All the procedures were carried out in accordance with the ethical guidelines and regulations. The unstimulated saliva samples were collected and assigned to four categories viz., (i) REP (ruptured ectopic pregnancy, n = 23); (ii) UREP (un-ruptured ectopic pregnancy, n = 17); (iii) PR (viable intra-uterine pregnancy, n = 15); and (iv) NPR (non-pregnant/control, n = 14). The mean age of the participants in the study was 27.47 ± 1.89 years for both ruptured and unruptured cases. In the case of pregnant and non-pregnant women, the mean age was 25.45 ± 2.96 years. Saliva collection was performed in the morning between 8 and 9 AM. Each volunteer was asked to abstain from food intake for 2 h before sampling and instructed for oral wash with sterile MilliQ water just before sampling. The saliva was collected by spitting method^48^ where the saliva was allowed to accumulate in the floor of the buccal cavity, and the volunteers spit out the saliva into sterile Falcon tubes every 60 s until the volume reached 2 mL. The collected samples were transported in a mini cooler box to the laboratory within 30 to 45 min after collection. An inhibitor cocktail was used to mitigate protease degradation during sample handling and transportation. The samples were then centrifuged at 4500×*g* for 15 min at 4 °C to remove the insoluble material and cells, if any, and then stored at − 80 °C until further use^49^.

### Protein precipitation

To precipitate the salivary proteins, TCA-acetone precipitation method^50^ was employed. First, the saliva sample was mixed with 10% TCA and 10 mM DTT in acetone, and allowed to incubate for an hour at − 20 °C. Subsequently, the samples were subjected to centrifugation at 5000×*g* at 4 °C for 20 min. The resulting pellets were washed twice with ice-cold acetone and centrifuged each time at 5000×*g* at 4 °C for 20 min. Finally, the pellets were air-dried and re-suspended in 50 mM Tris buffer (pH 8.8). The protein concentration was determined using Bradford method^51^.

### SDS-PAGE

To identify the salivary proteins, the samples were subjected to 12% SDS-PAGE analysis. A total of 30 μg of protein was taken from each individual sample and samples from each group were pooled separately. Subsequently, 30 μg of protein from the pooled salivary protein samples was mixed with sample buffer [50 mM Tris–HCl (pH 6.8), 2% SDS, 10% glycerol, 0.1% bromophenol blue, 100 mM β-mercaptoethanol] and heated at 60 °C for 1 min to ensure complete protein denaturation. The resultant mixture was loaded onto the gel, and electrophoresis was conducted at a constant current of 50 V. Following electrophoresis, the gels underwent a 2-min immersion in distilled water, followed by staining with 0.5% Coomassie Brilliant Blue (CBB) solution (composed of 40% methanol, 10% acetic acid, and 0.5% CBB R-250) at 37 °C for 2 h. Subsequently, a solution of 40% methanol and 10% acetic acid was used to de-stain the gels. Samples were pooled and quantified for group-wise REP, UREP, PR, and NPR, respectively, for LC–MS/MS analysis.

### In-gel trypsin digestion

Trypsin digestion for separation of proteins in gels was conducted according to Shevchenko et al.^52^. The protein bands in the gel were treated with acetonitrile to remove the stain. Then, the gels were sliced and subjected to reduction using dithiothreitol (DTT) and ammonium bicarbonate. Next, the gel pieces were alkylated with iodoacetamide, washed and then incubated with trypsin, overnight, at 37 °C. After digestion, the peptides were extracted using formic acid and acetonitrile. The extracted fractions were dried and stored for further LC–MS/MS analysis at − 20 °C.

### Peptide and protein identification by mass spectrometry

The peptides were desalted using C18 Pierce® Zip tips (Thermo Fisher Scientific, Waltham, MA, USA) and the digested peptide mixture was transferred to the vials in the LC autosampler. The peptides were ionized by nanospray capillary column (PepMap™ RSLC C18, Thermo Fisher Scientific, Waltham, MA, USA) and subjected to tandem mass spectrometry (MS/MS) on Q-Exactive HF (Thermo Fisher Scientific, Waltham, MA, USA). The threshold of false discovery rate was kept at 0.01. The MS/MS spectra were analyzed using Proteome Discoverer (Version 2.2) for protein identification. The MS/MS search was conducted using the SEQUEST search engine against the NCBI using *Homo sapiens* protein database. The search parameters included trypsin as a protease with one missed cleavage allowed. Carbamidomethyl cysteine was considered as a fixed modification, and oxidation of methionine was considered as a dynamic modification. The precursor ion mass error window was set at 10 ppm, and the fragment ion mass error window was set at 0.2 Da. To estimate the false discovery rate (FDR), peptide sequence analysis using a decoy database was enabled. High-confidence peptide identifications were obtained by setting a target FDR threshold of 1% at the peptide level.

### Functional annotation

The identified proteins (UniProtKB Accession) were subjected to functional enrichment analysis using databases. Briefly, comprehensive information about the evolution of protein-coding gene families (particularly, protein phylogeny), their function, and the impact due to genetic variation were characterized using PANTHER (v16.0) classification system. Venn diagram was created by using web-based visualization tools viz., ProteoRE (http://proteore.org/). The identified proteins were curated by database of pathways and reactions in human biology for frequent cross-referencing of other resources [NCBI, Ensembl, UniProt, KEGG (gene and compound), ChEBI, PubMed, and GO] using Reactome (https://reactome.org/PathwayBrowser/#TOOL=AT). The proteome Reactome-annotated data describe the possible reactions if all annotated proteins and small molecules are present in a cell and active simultaneously^53^. A binomial test calculates the probability for each result. The *p*-values were corrected for the multiple testing (Benjamini–Hochberg procedure) that arise from evaluating the submitted list of identifiers against every pathway^54^.

### Ethical statement

The study and the procedure followed in the sample collection were approved by the Institutional Human Ethics Committee (IHEC) (No. RNT/Stat/IEC/2019) of Ravindra Nath Tagore (RNT) Medical College, Udaipur.

## Results

### Total salivary proteome

**Figure 1:**
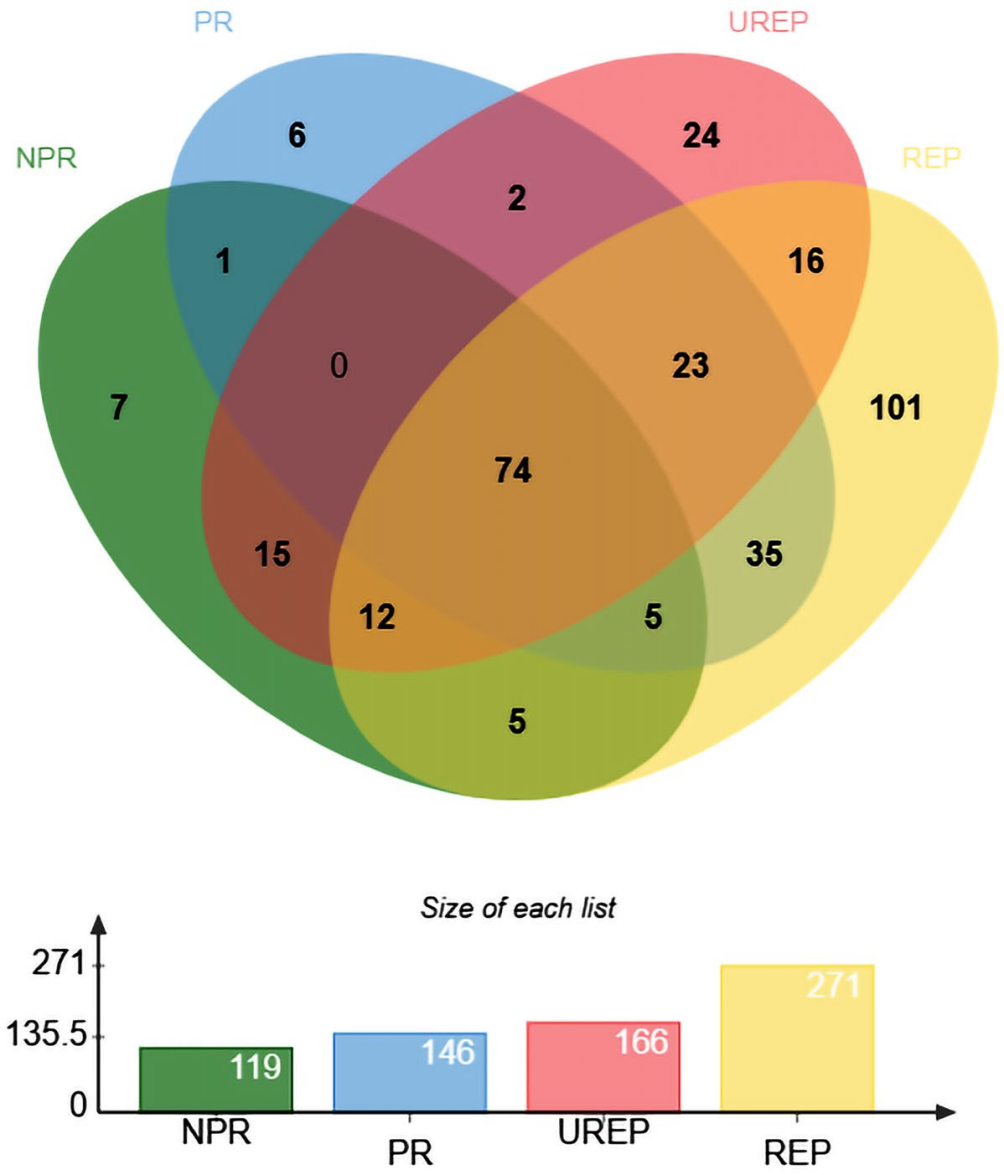
Representation of the salivary proteins identified in ruptured ectopic pregnancy, un-ruptured ectopic pregnancy, normal pregnancy, and non-pregnancy (REP, UREP, PR, NPR). Venn diagram was drawn using web-based visualization tools viz., ProteoRE (A) (http://proteore.org/).

The electrophoretically separated salivary proteins from various groups were subjected to mass spectrometry analysis (Fig. S1). A total of 326 proteins were identified across all groups of salivary samples. Among these 271, 166, 145 and 119 proteins were identified in REP, UREP, PR and NPR, respectively (Fig. 1). Further exploration of these 326 identified proteins, specifically 101 and 24 were found in REP (Table 1) and UREP (Table 2), respectively. In addition, 73 proteins were common to all four groups. Apart from REP and UREP, the proteins identified in the other groups are listed (Tables S1–S2).

**Table 1:** List of salivary proteins specific for ruptured ectopic pregnancy women (REP), identified by LC–MS/MS.

S.no.	UniprotAC^a^	Protein description^b^	Gene name^b^	AAs^b^	MW^b^	pI^b^	#Peptides	#Unique peptides
1.	**P0C0L5**	**Complement C4-B**	**C4B**	**1744**	**192,751**	**6.89**	**11**	**11**
2.	P07195	L-lactate dehydrogenase B chain	LDHB	334	36,638	5.71	5	4
3.	P40926	Malate dehydrogenase, mitochondrial	MDH2	338	35,503	8.92	4	4
4.	**O00391**	**Sulfhydryl oxidase 1**	**QSOX1**	**747**	**82,578**	**9.13**	**3**	**3**
5.	**P09960**	**Leukotriene A-4 hydrolase**	**LTA4H**	**611**	**69,285**	**5.8**	**3**	**3**
6.	P22735	Protein-glutamine gamma-glutamyltransferase K	TGM1	817	89,787	5.68	3	3
7.	P22894	Neutrophil collagenase	MMP8	467	53,412	6.38	3	3
8.	Q9UKR3	Kallikrein-13	KLK13	277	30,570	8.78	3	3
9.	P05120*	Plasminogen activator inhibitor 2	SERPINB2	415	46,596	5.46	2	2
10.	P15309	Prostatic acid phosphatase	ACPP	386	44,566	5.83	2	2
11.	P27695	DNA- (apurinic or apyrimidinic site) lyase	APEX1	318	35,555	8.33	2	2
12.	P36222	Chitinase-3-like protein 1	CHI3L1	383	42,625	8.69	2	2
13.	P52907	F-actin-capping protein subunit alpha-1	CAPZA1	286	32,923	5.45	2	2
14.	P61916	NPC intracellular cholesterol transporter 2	NPC2	151	16,570	7.57	2	2
15.	Q15782	Chitinase-3-like protein 2	CHI3L2	390	43,501	7.11	2	2
16.	Q6P4A8	Phospholipase B-like 1	PLBD1	553	63,255	9.11	2	2
17.	Q9NP55	BPI fold-containing family A member 1	BPIFA1	256	26,713	5.42	2	2
18.	P15311	Ezrin	EZR	586	69,413	5.94	3	1
19.	A0A087WSY6	Immunoglobulin kappa variable 3D-15	IGKV3D-15	115	12,534	5.14	2	1
20.	A0A0B4J1X8	Immunoglobulin heavy variable 3–43	IGHV3-43	118	13,077	5.28	2	1
21.	A0A0C4DH29	Immunoglobulin heavy variable 1–3	IGHV1-3	117	13,008	9.59	2	1
22.	P01602	Immunoglobulin kappa variable 1–5	IGKV1-5	117	12,782	8.49	2	1
23.	P01624	Immunoglobulin kappa variable 3–15	IGKV3-15	115	12,496	5.14	2	1
24.	P01743	Immunoglobulin heavy variable 1–46	IGHV1-46	117	12,933	9.1	2	1
25.	P01766	Immunoglobulin heavy variable 3–13	IGHV3-13	116	12,506	6.54	2	1
26.	P13797	Plastin-3	PLS3	630	70,811	5.41	2	1
27.	P13929	Beta-enolase	ENO3	434	46,987	7.58	2	1
28.	P19652	Alpha-1-acid glycoprotein 2	ORM2	201	23,603	5.03	2	1
29.	P23083	Immunoglobulin heavy variable 1–2	IGHV1OR15-1	117	13,085	9.23	2	1
30.	Q13162	Peroxiredoxin-4	PRDX4	271	30,540	5.86	2	1
31.	Q14651	Plastin-1	PLS1	629	70,253	5.28	2	1
32.	A0A075B6I4	Immunoglobulin lambda variable 10–54	IGLV10-54	117	12,395	7.85	1	1
33.	A0A075B6S5	Immunoglobulin kappa variable 1–27	IGKV1-27	117	12,712	8.5	1	1
34.	A0A0A0MS14	Immunoglobulin heavy variable 1–45	IGHV1-45	117	13,508	9.2	1	1
35.	A0A0B4J2D9	Immunoglobulin kappa variable 1D-13	IGKV1D-13	117	12,569	7.68	1	1
36.	O43307	Rho guanine nucleotide exchange factor 9	ARHGEF9	516	60,982	5.47	1	1
37.	O43451	Maltase-glucoamylase, intestinal	MGAM	1857	209,852	5.27	1	1
38.	O60218	Aldo–keto reductase family 1 member B10	AKR1B10	316	36,020	7.66	1	1
39.	O60635	Tetraspanin-1	TSPAN1	241	26,301	5.12	1	1
40.	O75368	SH3 domain-binding glutamic acid-rich-like protein	SH3BGRL	114	12,774	5.22	1	1
41.	P00390	Glutathione reductase, mitochondrial	GSR	522	56,257	8.74	1	1
42.	P00505	Aspartate aminotransferase, mitochondrial	GOT2	430	47,518	9.14	1	1
43.	P01040	Cystatin-A	CSTA	98	11,006	5.38	1	1
44.	P01704	Immunoglobulin lambda variable 2–14	IGLV2-14	120	12,597	6	1	1
45.	P01709	Immunoglobulin lambda variable 2–8	IGLV2-8	118	12,382	5.59	1	1
46.	P02750	Leucine-rich alpha-2-glycoprotein	LRG1	347	38,178	6.45	1	1
47.	P02751	Fibronectin	FN1	2477	272,320	5.32	1	1
48.	P02774	Vitamin D-binding protein	GC	474	52,918	5.32	1	1
49.	P04114	Apolipoprotein B-100	APOB	4563	515,605	6.58	1	1
50.	P04179	Superoxide dismutase [Mn], mitochondrial	SOD2	222	24,750	8.35	1	1
51.	P04792	Heat shock protein beta-1	HSPB1	205	22,783	5.98	1	1
52.	P05155*	Plasma protease C1 inhibitor	SERPING1	500	55,154	6.09	1	1
53.	P06865	Beta-hexosaminidase subunit alpha	HEXA	529	60,703	5.04	1	1
54.	P08571	Monocyte differentiation antigen CD14	CD14	375	40,076	5.84	1	1
55.	P09972	Fructose-bisphosphatealdolase C	ALDOC	364	39,456	6.41	1	1
56.	P14174	Macrophage migration inhibitory factor	MIF	115	12,476	7.73	1	1
57.	P15328	Folate receptor alpha	FOLR1	257	29,819	8.3	1	1
58.	P15814	Immunoglobulin lambda-like polypeptide 1	IGLL1	213	22,963	10.1	1	1
59.	P16070	CD44 antigen	CD44	742	81,538	5.13	1	1
60.	P17213	Bactericidal permeability-increasing protein	BPI	487	53,900	9.41	1	1
61.	P20618	Proteasome subunit beta type-1	PSMB1	241	26,489	8.27	1	1
62.	P22352	Glutathione peroxidase 3	GPX3	226	25,552	8.26	1	1
63.	P28827	Receptor-type tyrosine-protein phosphatase mu	PTPRM	1452	163,682	6.21	1	1
64.	P36871	Phosphoglucomutase-1	PGM1	562	61,449	6.3	1	1
65.	P36952	Serpin B5	SERPINB5	375	42,100	5.72	1	1
66.	P37288	Vasopressin V1a receptor	AVPR1A	418	46,800	9.48	1	1
67.	P38646	Stress-70 protein, mitochondrial	HSPA9	679	73,680	5.87	1	1
68.	P42658	Dipeptidylaminopeptidase-like protein 6	DPP6	865	97,588	5.95	1	1
69.	P46940	RasGTPase-activating-like protein IQGAP1	IQGAP1	1657	189,252	6.08	1	1
70.	P48637	Glutathione synthetase	GSS	474	52,385	5.67	1	1
71.	P51148	Ras-related protein Rab-5C	RAB5C	216	23,483	8.64	1	1
72.	P53634	Dipeptidyl peptidase 1	CTSC	463	51,854	6.53	1	1
73.	P54802	Alpha-N-acetylglucosaminidase	NAGLU	743	82,266	6.2	1	1
74.	P60900	Proteasome subunit alpha type-6	PSMA6	246	27,399	6.35	1	1
75.	P78324	Tyrosine-protein phosphatase non-receptor type substrate 1	SIRPA	504	54,967	6.51	1	1
76.	P78357	Contactin-associated protein 1	CNTNAP1	1384	156,267	6.61	1	1
77.	**P78417**	**Glutathione S-transferase omega-1**	**GSTO1**	**241**	**27,566**	**6.24**	**1**	**1**
78.	Q01082	Spectrin beta chain, non-erythrocytic 1	SPTBN1	2364	274,609	5.39	1	1
79.	Q05639	Elongation factor 1-alpha 2	EEF1A2	463	50,470	9.11	1	1
80.	Q08211	ATP-dependent RNA helicase A	DHX9	1270	140,958	6.41	1	1
81.	Q13231	Chitotriosidase-1	CHIT1	466	51,681	6.55	1	1
82.	Q13490	Baculoviral IAP repeat-containing protein 2	BIRC2	618	69,900	6.27	1	1
83.	Q13634	Cadherin-18	CDH18	790	88,073	4.98	1	1
84.	Q15084	Protein disulfide-isomerase A6	PDIA6	440	48,121	4.95	1	1
85.	Q16651	Prostasin	PRSS8	343	36,431	5.52	1	1
86.	Q16769	Glutaminyl-peptide cyclotransferase	QPCT	361	40,877	6.12	1	1
87.	Q5SRE7	Phytanoyl-CoA dioxygenase domain-containing protein 1	PHYHD1	291	32,411	5.88	1	1
88.	Q5TCS8	Adenylate kinase 9	AK9	1911	221,413	4.96	1	1
89.	Q6UWV7	Protein shisa-like-2A	FAM159A	190	20,306	4.94	1	1
90.	Q6ZN66	Guanylate-binding protein 6	GBP6	633	72,427	5.98	1	1
91.	Q86XD5	Protein FAM131B	FAM131B	332	35,769	4.34	1	1
92.	Q86YV5	Inactive tyrosine-protein kinase PRAG1	SGK223	1406	149,624	6.83	1	1
93.	Q8N556	Actin filament-associated protein 1	AFAP1	730	80,725	8.87	1	1
94.	Q8TAA3	Proteasome subunit alpha-type 8	PSMA8	256	28,530	9.07	1	1
95.	Q8WVC0	RNA polymerase-associated protein LEO1	LEO1	666	75,404	4.38	1	1
96.	Q8WWA0	Intelectin-1	ITLN1	313	34,962	5.66	1	1
97.	Q96G75	E3 ubiquitin-protein transferase RMND5B	RMND5B	393	44,414	6.15	1	1
98.	Q9BYZ2	L-lactate dehydrogenase A-like 6B	LDHAL6B	381	41,943	8.88	1	1
99.	Q9H0W9	Ester hydrolase C11orf54	C11orf54	315	35,117	6.23	1	1
100.	Q9UBR2	Cathepsin Z	CTSZ	303	33,868	6.7	1	1
101.	Q9UJ42	Probable G-protein coupled receptor 160	GPR160	338	39,787	8.84	1	1

^a^Proteins having at least one identified peptide in ectopic pregnancy saliva are listed with their UniprotKB accession numbers and length.

^b^Properties were retrieved using the PANTHER, DAVID and NCBI online database bioinformatics resource.

The proteins represent in bold letters have significant role in the ectopic pregnancy.

**Table 2:** List of salivary proteins specific for Unruptured ectopic pregnancy women (UREP) identified by LC–MS/MS.

S.no.	UniprotAC^a^	Protein description^b^	Gene Name^b^	AAs^b^	MW^b^	pI^b^	#Peptides	#Unique peptides
1.	P42357	Histidine ammonia-lyase (histidase)	HAL HIS	657	72,698	6.49	5	5
2.	P68104	Elongation factor 1-alpha 1	EEF1A1	462	50,141	9.10	3	3
3.	P19961	Alpha-amylase 2B	AMY2B	511	57,710	6.49	24	2
4.	Q9BYE4	Small proline-rich protein 2G (SPR-2G)	SPRR2G	73	8158	8.30	2	2
5.	P34931	Heat shock 70 kDa protein 1-like	HSPA1L	641	70,375	5.75	4	1
6.	P31947	14–3–3 protein sigma	SFN HME1	248	27,774	4.68	3	1
7.	A0A075B6K4	Immunoglobulin lambda variable 3–10	IGLV3-10	115	12,441	4.87	1	1
8.	A0A0C4DH72	Immunoglobulin kappa variable 1–6	IGKV1-6	117	12,697	8.01	1	1
9.	P03973	Antileukoproteinase (ALP)	SLPI	132	14,326	9.11	1	1
10.	P14735	Insulin-degrading enzyme	IDE	1019	117,968	6.16	1	1
11.	P26641	Elongation factor 1-gamma	EEF1G	437	50,119	6.27	1	1
12.	P47755	F-actin-capping protein subunit alpha-2 (CapZ alpha-2)	CAPZA2	286	32,949	5.58	1	1
13.	P59190	Ras-related protein Rab-15	RAB15	212	24,391	5.53	1	1
14.	P68363	Tubulin alpha-1B chain	TUBA1B	451	50,152	4.94	1	1
15.	Q13867	Bleomycin hydrolase (BH)	BLMH	455	52,562	5.87	1	1
16.	Q2LD37	Bridge-like lipid transfer protein family member 1	BLTP1	5005	555,482	6.12	1	1
17.	Q53RT3	Retroviral-like aspartic protease 1	ASPRV1	343	36,991	5.15	1	1
18.	Q86TJ2	Transcriptional adapter 2-beta	TADA2B	420	48,470	7.93	1	1
19.	Q8TER0	Sushi, nidogen and EGF-like domain-containing protein 1	SNED1	1413	152,204	6.46	1	1
20.	Q96FX8	p53 apoptosis effector related to PMP-22	PERP	193	21,386	6.68	1	1
21.	Q9NYC9	Dynein axonemal heavy chain 9	DNAH9	4486	511,877	5.64	1	1
22.	Q9NZT1	Calmodulin-like protein 5	CALML5	146	15,893	4.31	1	1
23.	Q9UBL6	Copine-7 (Copine VII)	CPNE7	633	70,294	5.97	1	1
24.	Q9UI42	Carboxypeptidase A4	CPA4	421	47,351	7.10	1	1

^a^Proteins having at least one identified peptide in ectopic pregnancy saliva are listed with their UniprotKB accession numbers and length.

^b^Properties were retrieved using the PANTHER, DAVID and NCBI online database bioinformatics resource.

### Functional annotation

**Figure 2:**
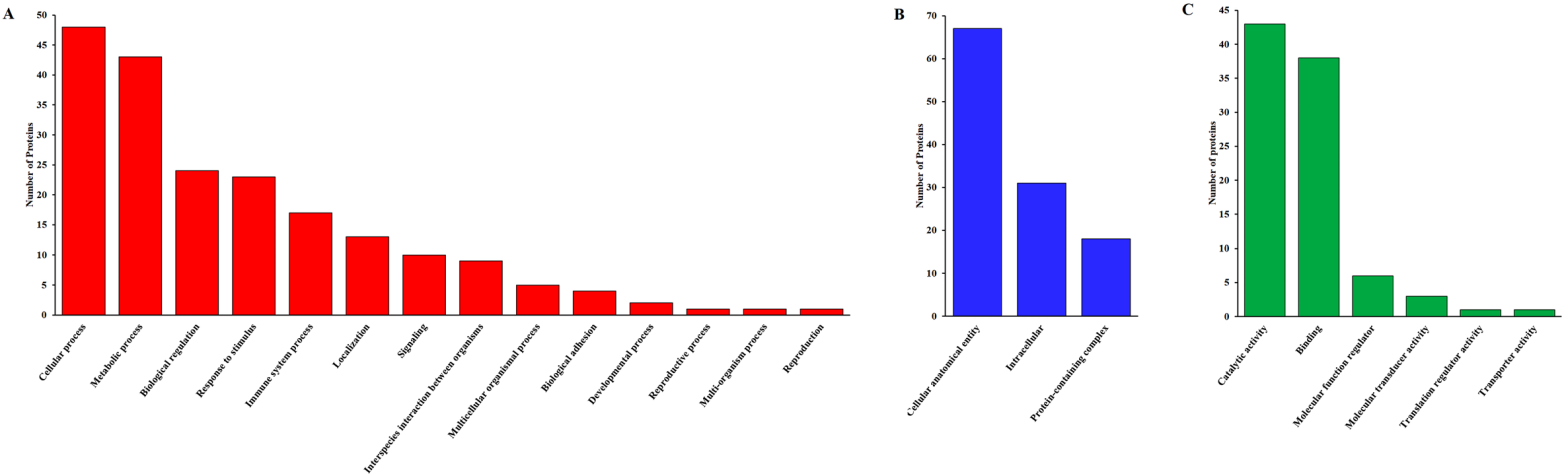
GO term for salivary proteins identified in REP condition. (**A**) Biological process, (**B**) Cellular component, (**C**) Molecular function.

The collected Uniprot GO terms by the identified proteins were subjected to functional annotation using PANTHER and Proteore databases. Proteins specific to REP were classified by their biological process, cellular components, and molecular function. Based on the biological process most of the proteins were related to cellular process (23.9%), whereas others were involved in metabolic processes (21.4%), biological regulation (11.9%), response to stimuli (11.4%), and immune system processes (8.5%), localization, signaling, interspecies interaction, multicellular organismal process, biological adhesion, developmental process, reproductive process, multi-organism process, and reproduction. Based on the cellular components, most of the salivary proteins got assigned to cellular anatomical entity (57.8%), intracellular (26.7%), and protein-containing complex (15.5%), respectively. According to the molecular function, the most prevalence was catalytic activity (46.7%), and binding (41.3%), followed by molecular function regulation, molecular transducer activity, translation regulator activity, and transporter activity, respectively (Fig. 2).

### Pathway enrichment analysis

**Figure 3:**
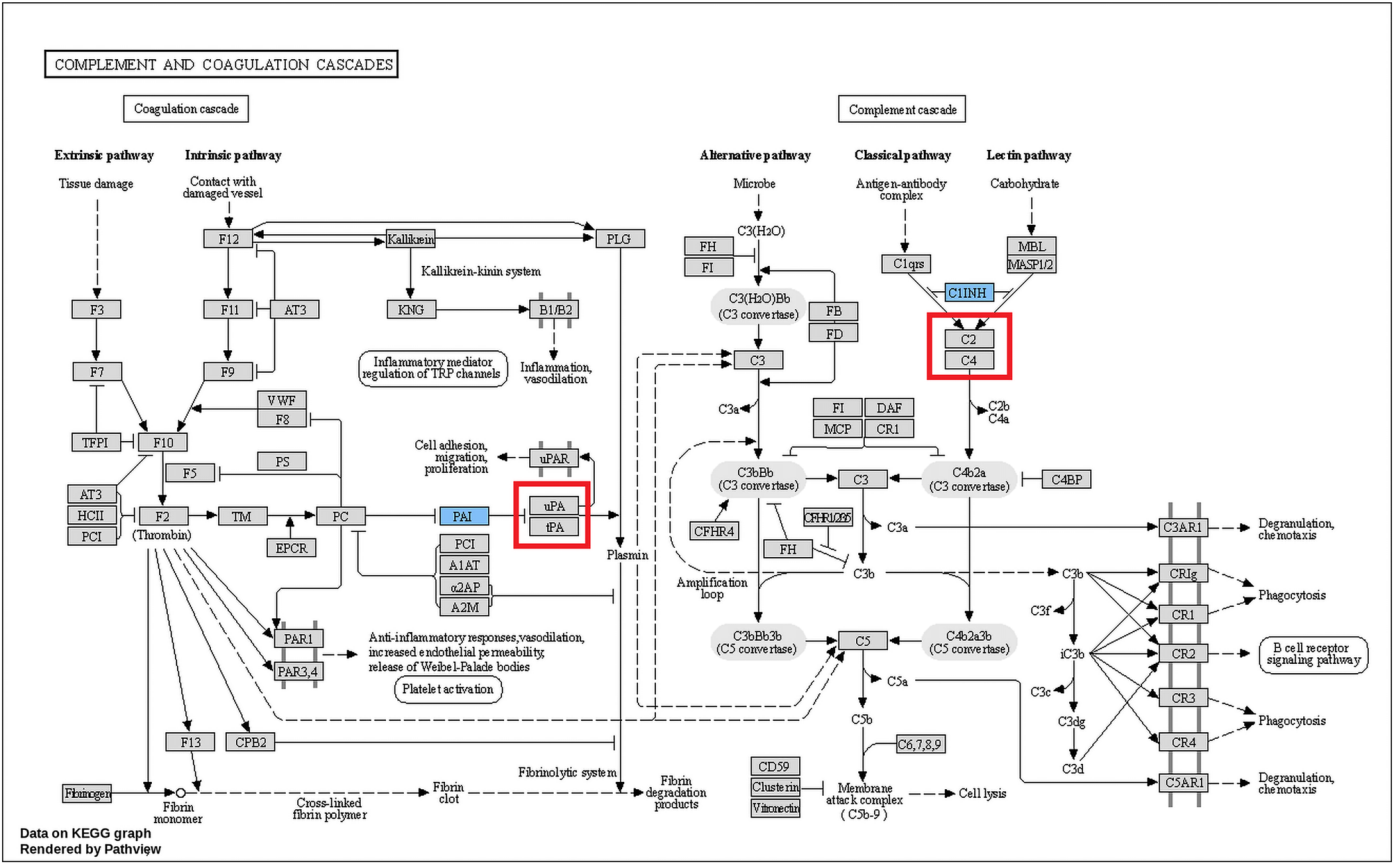
Pathway of complement and coagulation cascades with respect to identified REP-specific salivary proteins by KEGG (hsa04610). The blue color indicates the identified proteins, and the red color indicates the proteins associated with the pathways.

Reactome analysis was performed using the resources from NCBI, Ensembl, UniProt, KEGG, ChEBI, PubMed, and GO, which showed 81 out of the 101 identified proteins to be found in Reactome knowledge, where 485 pathways were hit at least once. About 25 significant pathways were sorted with *p*-value (Table S3). Further analysis revealed that two proteins, plasminogen activator inhibitor 2 (SERPINB2) and plasma protease C1 inhibitor (SERPING1) are specifically involved in coagulation and complement cascade pathways, respectively (Fig. 3).

### Protein–protein interaction

**Figure 4:**
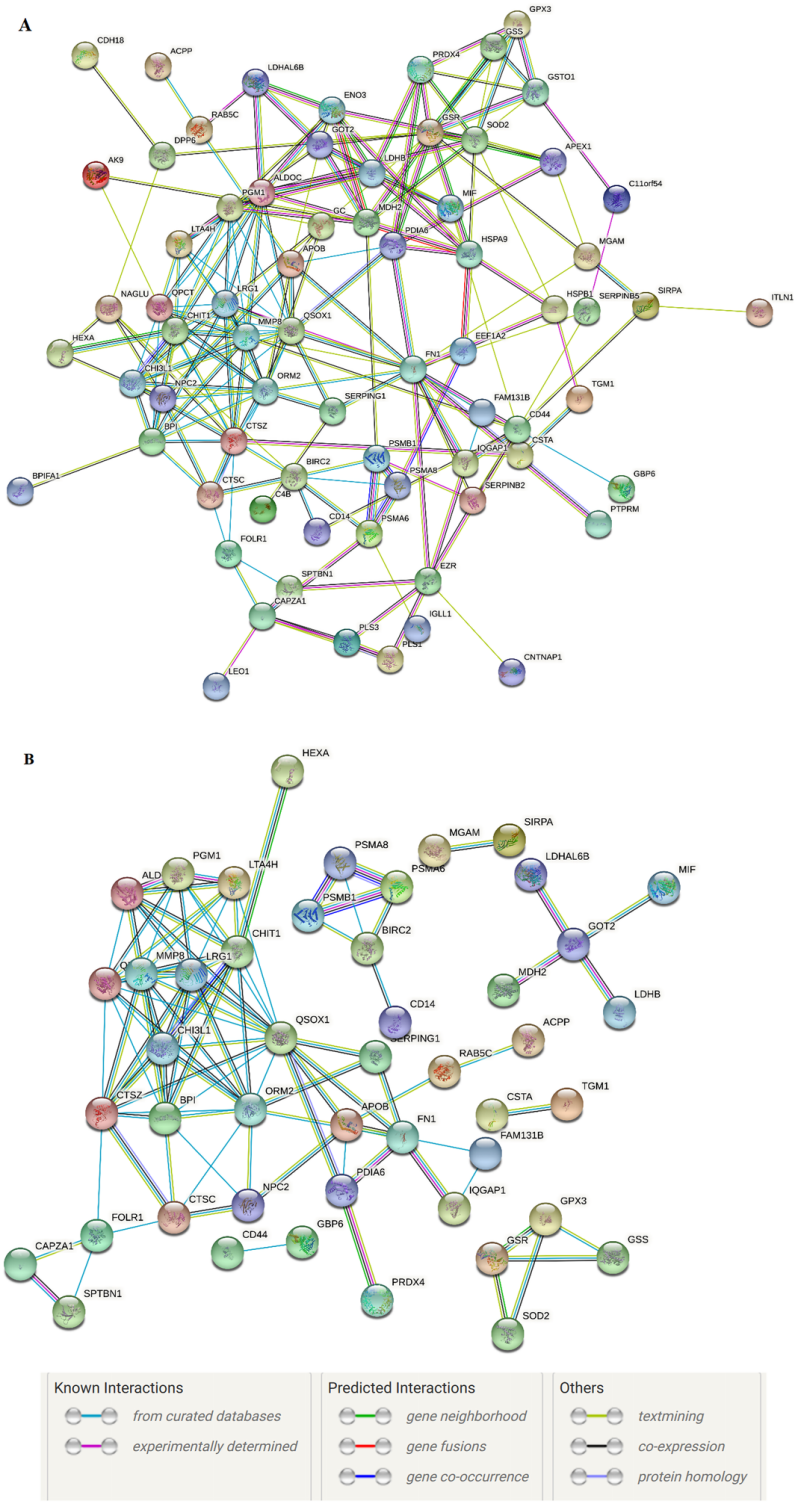
String representative network of the identified REP-specific salivary proteins. (**A**) The minimum required interaction score is set as 0.4. A total of 87 genes were connected with 204 paired relationships annotated. (**B**) The minimum required interaction score was set as 0.9. A total of 87 genes were related to 102 paired relationships. The PPI enrichment *p*-value: < 1.0e−16. No relationship nodes were removed from the network.

Protein–protein interaction network was constructed by retrieving the String, and the results showed that 87 proteins were in connection with other proteins, which lead to 204 paired relationships in medium interaction score, which indicates moderate confidence in the detected protein–protein interactions. Additionally, the proteins related to 102 relationships in high interaction score (Fig. 4), which are also part of the medium scoring pairs. The scores were calculated based on the data collected from experimental computational prediction with a confidence value. Among the proteins, QSOX1, CHI3L1, CHIT1, CTSZ, MMP8, PGM1, GSTO1, FN1 and LTA4H showed strong interactions among themselves and also with other proteins in the network.

## Discussion

In the present study, numerous immunological and non-immunological proteins have been detected in the saliva of women representing different conditions viz., REP (ruptured ectopic pregnancy; Group 1), UREP (un-ruptured ectopic pregnancy; Group 2), PR (pregnancy; Group 3) and NPR (non-pregnancy, Group 4). Specifically, REP saliva contains many defensive proteins which throw new light on the understanding of the defense mechanisms mediated by saliva during ectopic pregnancy. Also, these proteins have been reported to possess antibacterial activity^55^.

Fibronectin (FN1) is a protein found in the extracellular matrix (ECM) that regulates cell adhesion, spreading, migration, proliferation, and apoptosis^56^. In pregnant women, fetal fibronectin (fFN) plays an important role in the pathogenesis of preterm labor and premature rupture of fetal membranes^57,58^. Possibly, one of the salivary proteins in this study is fFN that has earlier been identified in the saliva of exclusively EPR cases. The expression of vitamin D-binding protein is lower in the placenta^59^ and has been found in serum^60^, leading to spontaneous miscarriages and preterm deliveries, respectively. Interestingly, in the present study vitamin D shows up in the salivary samples at REP. Another protein, known as apolipoprotein B-100 (ApoB), the level of which is higher in preeclampsia (PE), fetal growth restriction (FGR), and PE + FGR than the normal pregnancy^61^. Fibronectin, vitamin D-binding protein, and ApoB are found in saliva specifically during the REP which reflects that saliva has the potential to showcase the progress in ectopic pregnancy condition. The protein ezrin is identified in REP condition, which is an interesting observation since Ezrin and its activated form have been observed in endometriotic lesions, evidencing that these proteins could play some role in the migration and attachment of endometriotic lesions^62^.

Human saliva contains several Cystatin family proteins, including Cystatin-A, Cystatin-B, Cystatin-C, Cystatin-D, Cystatin-S, Cystatin-SA and Cystatin-SN^63^. Particularly appearance of Cystatin-S, a cysteine protease inhibitor, is prominent during the ovulatory phase^28^. However, in the present study, Cystatin-A appeared during REP condition. Several proteins, such as Alpha-1-acid glycoprotein 2, Complement C4-B, Immunoglobulin heavy variable 1–2, Immunoglobulin heavy variable 1–3, Immunoglobulin heavy variable 1–46, Prostatic acid phosphatase, and Vitamin D-binding protein have been identified in the human follicular fluid^64^. Interestingly, these proteins are present in the saliva of women during REP condition and completely absent in PR and NPR saliva.

Previous research has provided valuable insights into the immune mechanisms involved in reproductive health, shedding light on the role of the immune system in the context of fallopian tubes^65^. Additionally, Wicherek et al.^66,67^ reported increased levels of CD56+ and CD3+ cells, as well as heightened CD69 staining, in ruptured ectopic pregnancies. These findings suggest immune cell involvement in the process of tubal rupture, which is crucial information for understanding the pathogenesis of ectopic pregnancies. Furthermore, Visser et al.^68^ discussed the historical use of anti-Rh(D) immunoglobulin in Rh-negative women as a preventive measure against sensitization. This emphasizes the importance of immune-related interventions in reproductive health management. However, it is noteworthy that the current evidence on immune cell subtypes in tubal pathologies remains inconsistent. Only limited conclusions can be drawn at this stage, emphasizing the necessity for further research to explore and clarify the role of specific immune cell subtypes in tubal pathologies.

Notably, Complement C4-B is reportedly upregulated in the follicular fluid of patients with recurrent spontaneous abortion^69^, and its potential presence in saliva may have implications for ectopic pregnancy (EP) research. Additionally, proteins such as Plasminogen Activator Inhibitor 2 and 1, expressed in human saliva, may also be relevant to EP. The low expression rate of plasminogen activator inhibitor type-2 (PAI-2) leads to an increased risk of growth restriction in developing intrauterine layers^70^.

The pathways enrichment encoding genes-associated biological pathways are concerned with immunity, inflammation, homeostasis, and development. Importantly, plasminogen activator inhibitor 2 (SERPINB2) and plasma protease C1 inhibitor (SERPING1) are involved in coagulation and complement cascade which may throw open use as a putative salivary biomarker protein for diagnosis of REP condition. It has been emphasized that preeclampsia and eclampsia are two pregnancy complications that are basic to occurrence of REP is the presence of placental tissue in the maternal body and it is postulated that poor placentation results from inappropriate uterine spiral artery invasion^65^. The low level of SERPINB2 is associated with placental insufficiency, and the high level of tissue plasminogen activator is connected to endothelial dysfunction in patients with severe preeclampsia^71^. Low-level plasma protease C1 inhibitor has been used as a biomarker for preeclampsia^72^. The present study also finds that salivary proteins could be potential biomarkers of ectopic pregnancy, a subject worthy of further investigation.

Glutathione S-transferase omega-1 (GSTO1) is an enzyme that plays an important role in detoxification of environmental pollution and in xenobiotic metabolism. Also, the level of GSTO1 is related to fetal intrauterine growth restriction. Glutathione S-transferase omega-1 has been identified as a potential biomarker in a data-independent acquisition (DIA) proteomics study to evaluate ectopic pregnancy (EP) using serum samples. However, the study found that GSTO1 levels were significantly different between the intrauterine pregnancy (IP) and EP groups^47^. Functional annotation revealed that the salivary proteins of interest as above are majorly associated with binding property and regulatory function in REP and UREP conditions. Regulatory proteins activate or repress transcription. Activators enhance RNA polymerase interaction with the promoter, while repressors impede progress. These proteins are crucial for proper organ development by turning on specific genes at the right time. Protein–protein interaction analysis shows a crosstalk among QSOX1, CHI3L1, CHIT1, CTSZ, MMP8, PGM1, GSTO1, FN1, and LTA4H. These interactions have a significant role during miscarriage. Based on the reports these nine proteins are predominantly associated with ectopic pregnancy, which is recaptured in the present study.

Considering other regular body fluids, saliva has potential as a source of biomarker proteins. Since there are no remarkable reports on proteomic studies to show the variation of proteins present in saliva with respect to ectopic pregnancy condition by adopting proteins mass spectrometry technology, to the best of our knowledge, this is the first mass-spectrometry-based proteomic study of saliva to explore the salivary proteins focusing on ectopic pregnancy condition. Among the identified 271 proteins, the 101 REP-specific proteins offer the potential to spot out the promising protein biomarkers. Further studies on the immunoglobulin, fibronectin, vitamin D-binding protein, plasminogen activator inhibitor 2, ApoB, and Cystatin-A can potentially lead to selection of putative candidate salivary biomarkers of REP condition in women. Further, the functional annotation of salivary proteins strongly indicates that the proteins of interest are mostly extracellular proteins that participate in regulatory functions during REP and UREP conditions. Ectopic pregnancy, being a tissue-specific risk factor, identification of the panel of protein markers in the saliva may help in development of non-invasive/cost-affordable diagnostic tool for the EP. Moreover, our preliminary data suggests that salivary proteins, including Complement C4-B, Plasminogen Activator Inhibitor 2 and 1, and Glutathione S-transferase omega-1, hold promise as potential biomarkers for ectopic pregnancy. These proteins have been implicated in other issues in reproduction, and show potential relevance to ectopic pregnancy. A more elaborate and focused study, currently in progress, is expected to throw light on the specificity and diagnostic utility of the specific salivary protein(s) associated with ectopic pregnancy.

### Supplementary Information

Supplementary Information.

## Data Availability

The mass spectrometry proteomics data have been deposited to the ProteomeXchange Consortium via the PRIDE partner repository with the dataset identifier PXD036633.
