# Supplementary material for: Ectopic pregnancy: search for biomarker in salivary proteome
Source: Sci Rep. 2023 Oct 6;13:16828. doi: 10.1038/s41598-023-43791-7 (PMC10558548; doi:10.1038/s41598-023-43791-7)
Supplement: Supplementary file 1 — Supplementary Information. [file 41598_2023_43791_MOESM1_ESM.docx]

**Ectopic pregnancy: Search for biomarker in salivary proteome**

**Archunan Priya Aarthy^1,2,*^, Sangeetha Sen^1^, Mahalingam Srinivasan^3^, Subramanian Muthukumar^4,*^, Pakirisamy Madhanraj^5^, Mohammad Abdulkader Akbarsha^6,7^, Govindaraju Archunan^3,8,*^**

^1^Department of Obstetrics and Gynaecology, Rabindra Nath Tagore Medical College, Udaipur, Rajasthan, India.

^2^Department of Obstetrics and Gynaecology, Saveetha Medical College and Hospital, Deemed University, Chennai, India.

^3^Department of Animal Science, Bharathidasan University, Tiruchirappalli, Tamil Nadu, India.

^4^Deparment of Biotechnology, School of Chemical & Biotechnology (SCBT), SASTRA Deemed University, Thanjavur, Tamil Nadu, India.

^5^ Departmeny of Microbiology, Marudupandiyar College, Thanjavur, Tamil Nadu, India.

^6^Mahatma Gandhi-Doerenkamp Centre for Alternatives, Bharathidasan University, Tiruchirappalli, India. ^7^Department of Biotechnology & Microbiology, National College (Autonomous), Tiruchirappalli, India.

^8^Dean –Reseaech, Marudupandiyar College, Thanjavur, Tamil Nadu, India
**^*^Corresponding author**

**Table S.1:** List of salivary proteins specific for Pregnancy women (PR) analyzed by LC-MS/MS.

| ***S.No*** | ***UniprotAC^a^*** | ***Protein Description^b^*** | ***Gene Name^b^*** | ***AAs^b^*** | ***MW^b^*** | ***pI^b^*** | ***#Peptides*** | ***#Unique peptides*** |
| --- | --- | --- | --- | --- | --- | --- | --- | --- |
|  | P19021 | Peptidyl-glycine alpha-amidating monooxygenase | PAM | 973 | 108332 | 5.83 | 3 | 3 |
|  | A6NMY6 | Putative annexin A2-like protein | ANXA2P2 | 339 | 38659 | 6.48 | 2 | 2 |
|  | P07686 | Beta-hexosaminidase subunit beta | HEXB | 556 | 63137 | 5.90 | 2 | 2 |
|  | P21926 | CD9 antigen | CD9 | 228 | 25416 | 7.14 | 2 | 2 |
|  | Q8WUM4 | Programmed cell death 6-interacting protein | PDCD6IP | 868 | 96023 | 6.14 | 2 | 2 |
|  | Q9UHL4 | Dipeptidyl peptidase 2 | DPP7 | 492 | 54341 | 5.57 | 2 | 2 |

**Table S.2:** List of salivary proteins specific for Non pregnancy women (NPR) analyzed by LC-MS/MS.

| ***S.No*** | ***UniprotAC^a^*** | ***Protein Description^b^*** | ***Gene Name^b^*** | ***AAs^b^*** | ***MW^b^*** | ***pI^b^*** | ***#Peptides*** | ***#Unique peptides*** |
| --- | --- | --- | --- | --- | --- | --- | --- | --- |
|  | A0JNW5 | Bridge-like lipid transfer protein family member 3B | BLTP3B | 1464 | 164199 | 5.89 | 1 | 1 |
|  | P49368 | T-complex protein 1 subunit gamma | CCT3 | 545 | 60534 | 6.10 | 1 | 1 |
|  | P62258 | 14-3-3 protein epsilon | YWHAE | 255 | 29174 | 4.63 | 1 | 1 |
|  | Q01974 | Tyrosine-protein kinase transmembrane receptor ROR2 | ROR2 | 943 | 104757 | 5.90 | 1 | 1 |
|  | Q6UXH8 | Collagen and calcium-binding EGF domain-containing protein 1 | CCBE1 | 406 | 44103 | 6.72 | 1 | 1 |
|  | Q96A08 | Histone H2B type 1-A | H2BC1 | 127 | 14167 | 10.32 | 1 | 1 |
|  | Q9UKK3 | Protein mono-ADP-ribosyltransferase PARP4 | PARP4 | 1724 | 192595 | 5.43 | 1 | 1 |

^a^Proteins having at least one identiﬁed peptide in ectopic pregnancy saliva are listed with their UniprotKB accession numbers and length.

^b^Properties were retrieved using the PANTHER, DAVID and NCBI online database bioinformatics resource.

**Table S.3:** List of pathways exhibited by the salivary proteins identified during EPR, sorted by *p*-value (Accessed on 05 ‎June, ‎2021)

| **S. No.** | **Pathway name** | **Entities** | | | | **Interactors** | |
| --- | --- | --- | --- | --- | --- | --- | --- |
|  |  | **#found/ total** | **ratio** | **p-value** | **FDR*** | **# found/ total** | **ratio** |
|  | Innate Immune System | 41/1333 | 0.092 | 1.11E-16 | 5.58E-14 | 125/708 | 0.053 |
|  | Neutrophil degranulation | 24/480 | 0.033 | 3.22E-14 | 8.08E-12 | 9/10 | 7.45E-04 |
|  | Immune System | 47/2681 | 0.185 | 1.72E-10 | 2.87E-08 | 169/1621 | 0.121 |
|  | Cell surface interactions at the vascular wall | 13/257 | 0.018 | 3.60E-08 | 4.50E-06 | 7/65 | 0.005 |
|  | FCERI mediated NF-kB activation | 10/175 | 0.012 | 5.02E-07 | 5.02E-05 | 2/19 | 0.001 |
|  | Regulation of Complement cascade | 9/139 | 0.01 | 6.93E-07 | 5.75E-05 | 17/42 | 0.003 |
|  | CD22 mediated BCR regulation | 7/72 | 0.005 | 9.01E-07 | 6.19E-05 | 3/4 | 2.98E-04 |
|  | Signaling by the B Cell Receptor (BCR) | 10/189 | 0.013 | 9.99E-07 | 6.19E-05 | 14/44 | 0.003 |
|  | Complement cascade | 9/156 | 0.011 | 1.78E-06 | 9.76E-05 | 26/71 | 0.005 |
|  | Initial triggering of complement | 8/120 | 0.008 | 2.39E-06 | 1.20E-04 | 6/21 | 0.002 |
|  | Classical antibody-mediated complement activation | 7/97 | 0.007 | 6.29E-06 | 2.77E-04 | 2/2 | 1.49E-04 |
|  | Fc epsilon receptor (FCERI) signaling | 10/235 | 0.016 | 6.76E-06 | 2.77E-04 | 13/63 | 0.005 |
|  | FCGR activation | 7/103 | 0.007 | 9.26E-06 | 3.24E-04 | 6/6 | 4.47E-04 |
|  | Antigen activates B Cell Receptor (BCR) leading to generation of second messengers | 7/103 | 0.007 | 9.26E-06 | 3.24E-04 | 10/25 | 0.002 |
|  | Scavenging of heme from plasma | 7/106 | 0.007 | 1.11E-05 | 3.43E-04 | 1/12 | 8.94E-04 |
|  | Hemostasis | 18/801 | 0.055 | 1.15E-05 | 3.43E-04 | 34/334 | 0.025 |
|  | Role of LAT2/NTAL/LAB on calcium mobilization | 7/107 | 0.007 | 1.18E-05 | 3.43E-04 | 2/7 | 5.21E-04 |
|  | Gene and protein expression by JAK-STAT signaling after Interleukin-12 stimulation | 6/73 | 0.005 | 1.44E-05 | 3.88E-04 | 6/36 | 0.003 |
|  | Creation of C4 and C2 activators | 7/111 | 0.008 | 1.49E-05 | 3.88E-04 | 2/8 | 5.96E-04 |
|  | Binding and Uptake of Ligands by Scavenger Receptors | 8/167 | 0.011 | 2.57E-05 | 6.42E-04 | 15/33 | 0.002 |
|  | FCERI mediated MAPK activation | 7/124 | 0.009 | 3.01E-05 | 6.90E-04 | 2/20 | 0.001 |
|  | Interleukin-12 signaling | 6/84 | 0.006 | 3.14E-05 | 6.90E-04 | 6/56 | 0.004 |
|  | Role of phospholipids in phagocytosis | 7/129 | 0.009 | 3.86E-05 | 7.72E-04 | 5/12 | 8.94E-04 |
|  | FCERI mediated Ca+2 mobilization | 7/129 | 0.009 | 3.86E-05 | 7.72E-04 | 2/11 | 8.19E-04 |
|  | Interleukin-12 family signaling | 6/96 | 0.007 | 6.53E-05 | 0.001 | 6/114 | 0.008 |

# Number of identities

* False Discovery Rate

Figure S1. SDS-PAGE for Salivary proteiomic profiling


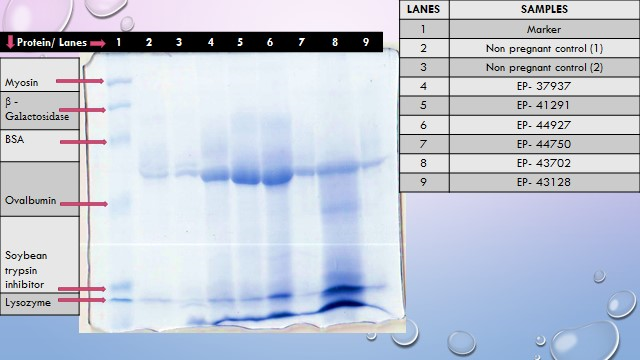


| Lanes | Description |
| --- | --- |
| 1 | Protein marker |
| 2 | Non Pregnant - NPR |
| 3 | Pregnant - PR |
| 4 | Unruptured ectopic pregnant - UREP |
| 5 | Ruptured ectopic pregnant - REP |
| 6 | Ruptured ectopic pregnant - REP |

Method of Q Exactive

| OVERALL METHOD SETTINGS |  |
| --- | --- |
| Global Settings |  |
| Use lock masses | best |
| Lock mass injection | Full MS |
| Chrom. peak width (FWHM) | 6 s |
| Time Method duration | 60.00 min |
| Customized Tolerances (+/-) |  |
| Lock Masses | ― |
| Inclusion | ― |
| Exclusion | ― |
| Neutral Loss | ― |
| Mass Tags | ― |
| Dynamic Exclusion | ― |

Experiment

| FULL MS / DD-MS² (TOPN) |  |
| --- | --- |
| General |  |
| Runtime | 0 to 60 min |
| Polarity | positive |
| In-source CID | 0.0 eV |
| Default charge state | 2 |
| Inclusion | ― |
| Exclusion | ― |
| Tags | ― |
| Full MS |  |
| Microscans | 1 |
| Resolution | 70,000 |
| AGC target | 3e6 |

Maximum IT 100 ms

Number of scan ranges 1

Scan range 400 to 1750 m/z

Spectrum data type Profile

dd-MS² / dd-SIM

Microscans 1

Resolution 17,500

AGC target 1e5

Maximum IT 100 ms

Loop count 10

MSX count 1

TopN 10

Isolation window 2.0 m/z

Isolation offset 0.0 m/z

Scan range 200 to 2000 m/z

Fixed first mass 50.0 m/z

(N)CE / stepped (N)CE nce: 29 Spectrum data type Profile

dd Settings

Minimum AGC target 1.00e3

Intensity threshold 1.0e4

Apex trigger ―

Charge exclusion unassigned, 1, 7, 8, >8 Multiple charge states all

Peptide match preferred

Exclude isotopes on

Dynamic exclusion 15.0 s

If idle .. do not pick others

Setup TUNEFILES

General

Switch Count 0

Base Tunefile C:\Xcalibur\Tune files\ESC-QE-nanotune-180418.mstune

CONTACT CLOSURE

General

Used True Start in Closed False Switch Count 0

SYRINGE

General

Used False Start in OFF True Stop at end of run False Switch Count 0

Pump setup

Syringe type Hamilton Flow rate 3.000 µL/min Inner diameter 2.303 mm Volume 250 µL

DIVERT VALVE A

General Used False Start in 1-2 True

Switch Count 0

DIVERT VALVE B

General

Used False Start in 1-2 True Switch Count 0

LOCK MASSES

1 entry Mass Polarity Start End Comment [m/z] [min] [min] 445.12003 Positive

Sample pickup:

Volume [µl] : 18.00 Flow [µl / min] : 5.00

Sample loading:

Volume [µl] : 20.00 Flow [µl / min] : 1.00 Max. pressure [Bar] : 400.00

Gradient:

Time [mm:ss] Duration [mm:ss] Flow [nl/min] Mixture [%B]

| 00:00 | 00:00 | 300 | 0 |
| --- | --- | --- | --- |
| 35:00 | 35:00 | 300 | 25 |
| 48:00 | 13:00 | 300 | 90 |
| 54:00 | 06:00 | 300 | 90 |
| 60:00 | 06:00 | 300 | 3 |

Pre-column equilibration: Volume [µl] : 0.00 Flow [µl / min] : 2.00 Max. pressure [Bar] : 350.00

Analytical column equilibration: Volume [µl] : 3.00

Flow [µl / min] : 1.00 Max. pressure [Bar] : 400.00

Auto-sampler wash:

Flush volume [µl] : 100.00

| === Tune Data: ===: |  |
| --- | --- |
| Spray Voltage (+): | 2200.00 |
| Spray Voltage (-): | 1500.00 |

Capillary Temperature (+ or +-): 305.00 Capillary Temperature (-): 250.00

Sheath Gas (+ or +-): 0.00

Sheath Gas (-): 0.00

Aux Gas (+ or +-): 0.00

Aux Gas (-): 0.00

Spare Gas (+ or +-): 0.00

Spare Gas (-): 0.00

Max Spray Current (+): 50.00 Max Spray Current (-): 50.00

Probe Heater Temp. (+ or +-): 350.00

Probe Heater Temp. (-): 350.00 S-Lens RF Level: 56.00

Ion Source: NSI

=== Calibration Data: ===: Mass Cal. (+) age (d): 4.90

Mass Cal. (-) age (d): 4.89

Isolation Cal. (+) age (d): 99.1

Isolation Cal. (-) age (d): 99.1 Amplifier Gain: 1000.000

Mass Calibration Parameter (0): 7.90000000e+01 Mass Calibration Parameter (1): 1.00000000e+02 Mass Calibration Parameter (2): 1.73279398e-09 Mass Calibration Parameter (3): 1.59220758e+09 Mass Calibration Parameter (4): -2.30194649e-12 Mass Calibration Parameter (5): 1.59220758e+09 Mass Calibration Parameter (6): -2.66331722e-10 Mass Calibration Parameter (7): 1.55126609e+09 Mass Calibration Parameter (8): 4.92801661e-13 Mass Calibration Parameter (9): 1.55126609e+09

Mass Calibration Parameter (10): -1.14000000e-06

Mass Calibration Parameter (11): 0.00000000e+00 Mass Calibration Parameter (12): -1.14000000e-06 Mass Calibration Parameter (13): 0.00000000e+00 Mass Calibration Parameter (14): -1.02106533e-12 Mass Calibration Parameter (15): 1.59220760e+09 Mass Calibration Parameter (16): -7.54030880e-13 Mass Calibration Parameter (17): 1.55126610e+09 Mass Calibration Parameter (18): -6.40724443e-11 Mass Calibration Parameter (19): 1.43098704e+09 Mass Calibration Parameter (20): -3.11636511e-11 Mass Calibration Parameter (21): 1.39090074e+09 Mass Calibration Parameter (22): 0.00000000e+00 Mass Calibration Parameter (23): 0.00000000e+00 Mass Calibration Parameter (24): 0.00000000e+00 Mass Calibration Parameter (25): 0.00000000e+00 Mass Calibration Parameter (26): 0.00000000e+00 Mass Calibration Parameter (27): 0.00000000e+00 Mass Calibration Parameter (28): 0.00000000e+00 Mass Calibration Parameter (29): 0.00000000e+00 Mass Calibration Parameter (30): 4.20000000e+01

Mass Calibration Parameter (31): 1.00000000e+00 Mass Calibration Parameter (32): 1.00000000e+00 Mass Calibration Parameter (33): 0.00000000e+00 Mass Calibration Parameter (34): 2.57694532e+01 Mass Calibration Parameter (35): 1.17147754e+06 Mass Calibration Parameter (36): 1.17147754e+05 Mass Calibration Parameter (37): 1.28000000e+03 Mass Calibration Parameter (38): 2.56000000e+02 Mass Calibration Parameter (39): 1.60034644e+09 Mass Calibration Parameter (40): 6.00000000e+00 Mass Calibration Parameter (41): 1.38066190e+02 Mass Calibration Parameter (42): 7.00989448e+02 Mass Calibration Parameter (43): 1.95087652e+02 Mass Calibration Parameter (44): 5.89713307e+02 Mass Calibration Parameter (45): 5.24264964e+02 Mass Calibration Parameter (46): 3.59733856e+02 Mass Calibration Parameter (47): 1.22199064e+03 Mass Calibration Parameter (48): 2.35626093e+02 Mass Calibration Parameter (49): 1.42197786e+03 Mass Calibration Parameter (50): 2.18429370e+02 Mass Calibration Parameter (51): 1.62196509e+03 Mass Calibration Parameter (52): 2.04520553e+02 Mass Calibration Parameter (53): 1.00000000e+00 Mass Calibration Parameter (54): 1.00000000e+00 Mass Calibration Parameter (55): 1.00000000e+00 Mass Calibration Parameter (56): 2.57422190e+01 Mass Calibration Parameter (57): 9.76791655e+05 Mass Calibration Parameter (58): 9.76791655e+04 Mass Calibration Parameter (59): 2.40000000e+03 Mass Calibration Parameter (60): 2.56000000e+02 Mass Calibration Parameter (61): 1.60034674e+09

Mass Calibration Parameter (62): 8.00000000e+00 Mass Calibration Parameter (63): 2.65147903e+02 Mass Calibration Parameter (64): 5.05903486e+02 Mass Calibration Parameter (65): 5.14284397e+02 Mass Calibration Parameter (66): 3.63252270e+02 Mass Calibration Parameter (67): 1.27999721e+03 Mass Calibration Parameter (68): 2.30252154e+02 Mass Calibration Parameter (69): 1.37999083e+03 Mass Calibration Parameter (70): 2.21753275e+02 Mass Calibration Parameter (71): 1.47998444e+03 Mass Calibration Parameter (72): 2.14131002e+02 Mass Calibration Parameter (73): 1.57997805e+03 Mass Calibration Parameter (74): 2.07244311e+02 Mass Calibration Parameter (75): 1.67997166e+03 Mass Calibration Parameter (76): 2.00982013e+02 Mass Calibration Parameter (77): 1.77996528e+03 Mass Calibration Parameter (78): 1.95255127e+02 Mass Calibration Parameter (79): 0.00000000e+00 Mass Calibration Parameter (80): 0.00000000e+00 Mass Calibration Parameter (81): 0.00000000e+00 Mass Calibration Parameter (82): 0.00000000e+00 Mass Calibration Parameter (83): 0.00000000e+00 Mass Calibration Parameter (84): 0.00000000e+00 Mass Calibration Parameter (85): 0.00000000e+00 Mass Calibration Parameter (86): 0.00000000e+00 Mass Calibration Parameter (87): 0.00000000e+00 Mass Calibration Parameter (88): 0.00000000e+00 Mass Calibration Parameter (89): 0.00000000e+00 Mass Calibration Parameter (90): 0.00000000e+00 Mass Calibration Parameter (91): 0.00000000e+00 Mass Calibration Parameter (92): 0.00000000e+00

Mass Calibration Parameter (93): 0.00000000e+00 Mass Calibration Parameter (94): 0.00000000e+00 Mass Calibration Parameter (95): 0.00000000e+00 Mass Calibration Parameter (96): 0.00000000e+00 Mass Calibration Parameter (97): 0.00000000e+00 Mass Calibration Parameter (98): 0.00000000e+00 Mass Calibration Parameter (99): 0.00000000e+00 Mass Calibration Parameter (100): 0.00000000e+00 Mass Calibration Parameter (101): 0.00000000e+00 Mass Calibration Parameter (102): 0.00000000e+00 Mass Calibration Parameter (103): 0.00000000e+00 Mass Calibration Parameter (104): 0.00000000e+00 Mass Calibration Parameter (105): 0.00000000e+00 Mass Calibration Parameter (106): 0.00000000e+00 Mass Calibration Parameter (107): 0.00000000e+00 Mass Calibration Parameter (108): 0.00000000e+00 Mass Calibration Parameter (109): 0.00000000e+00 Mass Calibration Parameter (110): 0.00000000e+00 Mass Calibration Parameter (111): 0.00000000e+00 Mass Calibration Parameter (112): 0.00000000e+00 Mass Calibration Parameter (113): 0.00000000e+00 Mass Calibration Parameter (114): 0.00000000e+00 Mass Calibration Parameter (115): 0.00000000e+00 Mass Calibration Parameter (116): 0.00000000e+00 Mass Calibration Parameter (117): 0.00000000e+00 Mass Calibration Parameter (118): 0.00000000e+00 Mass Calibration Parameter (119): 0.00000000e+00 CTCD Scale (+): 2.035

| CTCD Scale (-): | 3.884 |
| --- | --- |
| CTCD Para (+): | 0.800 |
| CTCD Para (-): | 0.500 |

Detect Delay: 5.50

Relais Delay: 5.00

Res.-Dep. Delay: 10.00

Quad DC (+,0): 3.600

Quad DC (+,1): 0.000

Quad DC (-,0): -4.772

Quad DC (-,1): 0.000

Quad OTK (+,0):-24.983

Quad OTK (+,1):-0.056

Quad OTK (-,0): 13.110

Quad OTK (-,1): 0.077

| Quad Para A: | 0.000 | |
| --- | --- | --- |
| Quad Para B: | 0.000 | |
| Quad Para C: | 0.000 | |
| CLT GND Voltage (+): | | 21.60 |
| CLT GND Voltage (-): | | 14.20 |
| CLT Offset Voltage (+): | | 1650.0 |
| CLT Offset Voltage (-): | | 1650.0 |
| CLT Push Voltage (+): | | 110.0 |
| CLT Push Voltage (-): | | 110.0 |
| CLT Pull Voltage (+): | | 280.0 |
| CLT Pull Voltage (-): | | 280.0 |
| Lens 6 Voltage (+): | | 670.0 |
| Lens 6 Voltage (-): | | 670.0 |
| Z-Lens 3 Voltage (+): | | 230.0 |
| Z-Lens 3 Voltage (-): | | 230.0 |
| De-Inject Voltage (+): | | 40.0 |
| De-Inject Voltage (-): | | 40.0 |

De-Measure Voltage (+): 470.0

De-Measure Voltage (-):470.0 CE-Inject Voltage (+): 3800.0

CE-Inject Voltage (-): 3800.0 LowSNQuanLevel: 1

=== Configuration Data: ===: Preamp Protect Mode: 0 TMP Sweep Mode: 1

Temp Sensor Mode: 0

=== Identification: ===:

SW Version: 2.9-290033/2.9.0.2926
